# Design and validation of a Questionnaire on the factors influencing self-care behaviors in patients with Multiple sclerosis (QFASMS)

**DOI:** 10.1186/s12883-023-03522-x

**Published:** 2024-01-04

**Authors:** Fahimeh Pourhaji, Jamshid Jamali, Mousa Mahdizadeh Taraghdar, Nooshin Peyman, Hadi Tehrani

**Affiliations:** 1 Department of Health Education and Health Promotion, Student Research Committee, Mashhad University of Medical Sciences, Mashhad, Iran; 2 Social Determinants of Health Research Center, Mashhad University of Medical Sciences, Mashhad, Iran; 3 Department of Health Education and Health Promotion, School of Health, Mashhad University of Medical Sciences, Mashhad, Iran; 4 Department of Biostatistics, School of Health, Mashhad University of Medical Sciences, Mashhad, Iran; 5 Department of Medical Surgical Nursing, School of Nursing and Midwifery, Mashhad University of Medical Sciences, Mashhad, Iran

**Keywords:** Multiple sclerosis, Self-care, Validity and reliability, Psychometrics

## Abstract

**Background:**

Multiple sclerosis (MS) is a chronic inflammatory autoimmune disease of the central nervous system (CNS). Since MS does not have a definitive cure, individuals affected by it need to adapt and coordinate with their chronic illness in order to fulfill their duties and responsibilities. The first step in helping patients to better care for and manage their illness is to engage in self-care behaviors. This study was conducted with the aim of design and validation of a questionnaire on the factors influencing self-care behaviors in patients with Multiple sclerosis.

**Methods:**

This cross-sectional study was conducted on Multiple sclerosis patients in Iran in 2023. The age range of patients varied between 22 and 52 years. Having MS disease, passing one year of the disease duration, living in Mashhad city, having informed consent to participate in the study and not completing the questionnaire were the entry and exit criteria of the study.

**Results:**

This study was conducted on 500 patients with multiple sclerosis. Based on the results of psychometrics (face, content and construct validity), the number of questions was reduced from 120 to 47 questions and 73 questions were eliminated. Finally, the questionnaire was approved with 47 questions and 4 subscales of understanding the symptoms of the disease (9 questions), tendency to conscious and targeted care (21 questions), laziness in care (8 questions) and tendency to receive therapy services (9 questions). Cronbach's alpha and McDonald's omega index for all questionnaire questions were 0.877 and 0.881, respectively.

**Conclusions:**

Based on the results of this questionnaire, 47 questions and 4 subscales can be used to measure the factors influencing the adoption of self-care behaviour’s in patients with multiple sclerosis.

**Supplementary Information:**

The online version contains supplementary material available at 10.1186/s12883-023-03522-x.

## Background

Multiple sclerosis (MS) is an autoimmune disease of the central nervous system (CNS) that causes physical and cognitive impairments in young adults [1]. Multiple sclerosis has no cure and its long-term outcome is unpredictable [2]. The recent estimates indicate that the prevalence of this disease is 2.8 million people worldwide [3]. The phenomenon of MS, along with its socioeconomic effects, is spreading worldwide [4]. Individuals with this disease need to adapt to its chronic challenges The progress of MS and the side effects of drugs demonstrate that pharmacological interventions alone are not sufficient for controlling the disease [5]. One of the ways that patients with MS can effectively adapt to the complications of the disease is self-care practices [6]. The results of a study showed that self-care empowerment is an effective method for coping with symptoms and signs of MS [7]. Self-care promotes the quality of life for individuals with MS [8].

The results of a study emphasize the significant role of self-care in improving the quality of life, sleep quality, self-esteem, and fatigue management in patients with MS [9]. Self-care activities can encourage individuals to maintain health and well-being, enhance their adaptability, reduce the degree of disability, and consequently reduce healthcare costs [10].

According to literature, numerous questionnaires have been designed and implemented regarding self-care. There is no comprehensive tool available in Iran and other countries to assess all factors affecting self-care in patients with multiple sclerosis. The available questionnaires only examined some aspects of self-care, such as self-care in activities of daily living (ADL) among people with multiple sclerosis (MS) [11].

In the formation of self-care behavior, a wide range of personal and environmental factors play a significant role, and paying attention to them can greatly contribute to disease management. A tool designed with a focus on these factors can assess the self-care behavior of patients with Multiple Sclerosis. Therefore, the aim of this study was to design and validation of a questionnaire on the factors influencing self-care behaviors in patients with Multiple sclerosis.

## Methods

This cross-sectional study was conducted on multiple sclerosis patients in Mashhad city, in 2023.

### Participants and recruitment

Participants were included in the study by simple random method. The participants were selected from among the patients referred to the Comprehensive MS Center and the MS Association, who were between the ages of 22 and 52 years. The participants were selected from different geographical areas, urban and rural, of both sexes and different ages. Data collection lasted from November 2022 to May 2023. After necessary arrangements with the officials, the necessary permits were obtained to enter the comprehensive center and the MS Association. The researcher followed the research by explaining the objectives and importance of the research to the qualified patients who agreed to cooperate.

### Inclusion and exclusion criteria

The inclusion criteria for all samples were 1) having MS disease, 2) passing one year of the disease duration according to the 2017 McDonald criteria [12], 3) Living in Mashhad city, 4) having full consent to participate in the research and 5) knowingly completing the written consent form. Exclusion criteria included failure to complete the questionnaire.

### Instruments

In this research, two demographic questionnaires and factors affecting self-care of multiple sclerosis patients (QFASMS) were used to collect data.

### The demographic questionnaire

This section was investigated with questions such as gender, age, education level, duration of illness, age of onset of illness and employment status.

### Questionnaire of Factors Affecting Self-Care in Multiple Sclerosis Patients (QFASMS)

This questionnaire has 47 questions and 4 subscales of understanding disease symptoms (9 questions), tendency to conscious and targeted care (21 questions), laziness in care (8 questions) and tendency to receive therapy services (9 questions). A 5-point Likert scale (strongly disagree = 1, disagree = 2, neither agree nor disagree = 3, agree = 4 and strongly agree = 5) was used to measure the questions. Questionnaire of factors affecting the self-care of patients with multiple sclerosis (QFASMS) and 4 subscales were created by the researcher for this study. An English language version was uploaded as a supplementary file.

### Design of instrument

#### Qualitative stage

In the qualitative phase, a grounded theory study was conducted among multiple sclerosis patients. The interviews were conducted by referring to the comprehensive center and the MS Association and continued until data saturation was reached. Finally, the data was collected by conducting 28 in-depth and unstructured interviews. Twenty-one interviews were conducted with patients, and 7 additional interviews were conducted with relatives, specialists, and individuals related to the patients. MAXQADA version 10 software was used to extract the codes from the interviews. The data analysis method in the qualitative phase has been described in detail in previous studies [3].

#### Quantitative stage

At this stage, a question bank was designed according to the concepts of qualitative research and literature review. Face validity (qualitative and quantitative), content validity (qualitative and quantitative) and construct validity (confirmatory factor analysis) were conducted to evaluate the psychometrics of the questionnaire. The reliability of the tool was evaluated using McDonald's omega coefficient, Cronbach's alpha coefficient and Interclass Correlation Index (ICC) value. It should be noted that data collection was done in the quantitative part by distributing questionnaires among the patients of the Comprehensive Center and the MS Association. Next, the details of these steps will be described:

### Face validity

In order to check the qualitative face validity, ten members of the expert panel (written and email) were selected by purposive sampling method. Also, to get the opinions of the target group (MS patients), six of them were interviewed. Their opinions were collected regarding the difficulty of understanding phrases and words, the appropriate fit and relationship of items, insufficient perceptions of the phrases or the existence of insufficiency in the meanings of the words.

Quantitative face validity was done by measuring the impact score [13]. In this way, the questions were given to 30 people from the target group. The patients were asked to evaluate each item in terms of importance and assign a score of 1 to 5 to each item according to the level of importance.

Thus, for each item of the instrument, the 5-part Likert scale is: completely important (score 5), important (score 4), moderately important (score 3), slightly important (score 2) and Not important at all (score 1) was considered. Then using the formula of the item impact method (Impact Score = Frequency (%) x Importance), face validity was calculated. Questions with a score of less than 1.5 were eliminated.

### Content validity

In order to qualitatively check the content of the questionnaire, 12 members of the expert panel were selected using the convenience method. The opinion of the experts was collected about the importance of questions, placement of questions, grammar, and word usage. Content validity ratio (CVR) and content validity index (CVI) were used to evaluate quantitative content validity [14].

In order to calculate CVR, the questionnaire was sent to 12 specialists (health education, neurologist and psychiatrist). The experts were asked to rate each item of the tool in three ranges: "necessary", "useful but not necessary" and "not necessary". Content validity ratio (CVR) was calculated using Law she’s formula (1) [15]. After determining and calculating CVR, the questionnaire was examined to calculate CVI. In this part, experts were asked to comment on the following three criteria based on a 4-point Likert scale: (relevance or specificity, simplicity and fluency, and clarity or transparency). CVI was calculated by dividing the number of experts who chose option 3 and 4 by the total number of experts [16].

1 $$CVR = \frac{{n}_{E}- N/2}{N/2}$$

### Construct validity

To check the validity of the construct, Confirmatory factor analysis (CFA) was used [17]. We checked whether the model fit the data collected from the sample. Using Mahalanobis distance statistics, outliers were investigated and outliers were removed if necessary. The normality of the explanation of the data was confirmed by Mardia's test [18]. Also, in order to estimate the parameters, the maximum likelihood method was used. Confirmatory factor analysis was performed using AMOS version 24 software. The questions that had a weak regression coefficient (factor loading) were removed from the questionnaire. The model was evaluated using fit indices of chi-square ratio to the degree of freedom (× 2/df < 5); comparative fit index (CFI > 0.9); root means the square error of approximation (RMSEA ≤ 0.08); Tucker-Lewis index (TLI < 0.8) [19]. The Hoelter test was used to determine the appropriate sample size the CFA. To evaluate the SEM sample size, Hoelter presented the Critical N (CN) statistic, where a CN ≥ 200 was appropriate [20, 21]. The data were collected and the SEM model was determined. The post-hoc sample power was estimated with the non-centrality parameter (NCP or λ) [21]. The value of NCP and Fmin was obtained from the model. The sample size (N) was calculated as: N = (NCP/Fmin) + g [20]. In this study, a minimum sample size of 233 participants, with an error rate of 0.05, was sufficient for the CFA. In this study, a sample size of 500 people was used to check construct validity [21].

### Reliability

In this research, two methods of McDonald's omega coefficient and Cronbach's alpha coefficient were used to evaluate the reliability of the questionnaire. The software SPSSv22 was used to calculate Cronbach's alpha coefficient and McDonald's omega coefficient. The results indicate that MacDonald's omega coefficient provides a more accurate reliability coefficient than Cronbach's alpha [22]. A reliability coefficient value of more than 0.70 is considered acceptable [23, 24]. To assess the reliability, the value of Intraclass correlation index (ICC) was measured using a two-week test–retest approach for a group of 30 MS patients [25]. To assess the reliability, the value of Interclass correlation index (ICC) was measured using a two-week test–retest approach for a group of 30 MS patients. Data were analyzed using a single rater/measurement, absolute-agreement, 2-way random effects model (ICC 2, 1). Values less than 0.5, between 0.5 and 0.75, between 0.75 and 0.9, and more than 0.90 based on the 95% confidence interval of the ICC estimate, indicate poor, moderate, good, and excellent reliability, respectively [26].

## Results

### The characteristics of the participants

This study was conducted on 500 patients with multiple sclerosis. In this section, the average (± standard deviation) age of the participants was 36.46 (± 7.39). The mean and standard deviation of the age of disease onset were 28.5 (± 6.61). The mean and standard deviation of the duration of the disease was 8.18 (± 5.43) (Table 1).

**Table 1:** Demographic characteristics of participants (*n* = 500)

Socio-demographic characteristics	n	(%)
**Age** (X ± SD)	36.46 ± 7.39	
**Gender**		
Female	351	70.2
Male	149	29.8
**Marital status**		
Single	94	18.8
Married	351	70.2
Isolated	52	10.4
Widow	3	0.6
**Education level**		
under diploma	82	16.4
Diploma	130	26.0
Associate Degree	42	8.4
Bachelor's degree	174	34.8
Master's degree and above	70	14
Doctorate	2	0.4
**Occupational status**		
Employee	71	14.2
Retired	23	4.6
Self-employment	81	16.2
Housewife	201	40.2
Worker	25	5
Unemployed	60	12
Other	39	7.8
**Place of residence**		
City	481	96.2
Village	19	3.8
**Duration of disease** (X ± SD)	8.18 ± 5.43	
**Onset Age** (X ± SD)	28.5 ± 6.61	

### Qualitative phase

**Fig. 1:**
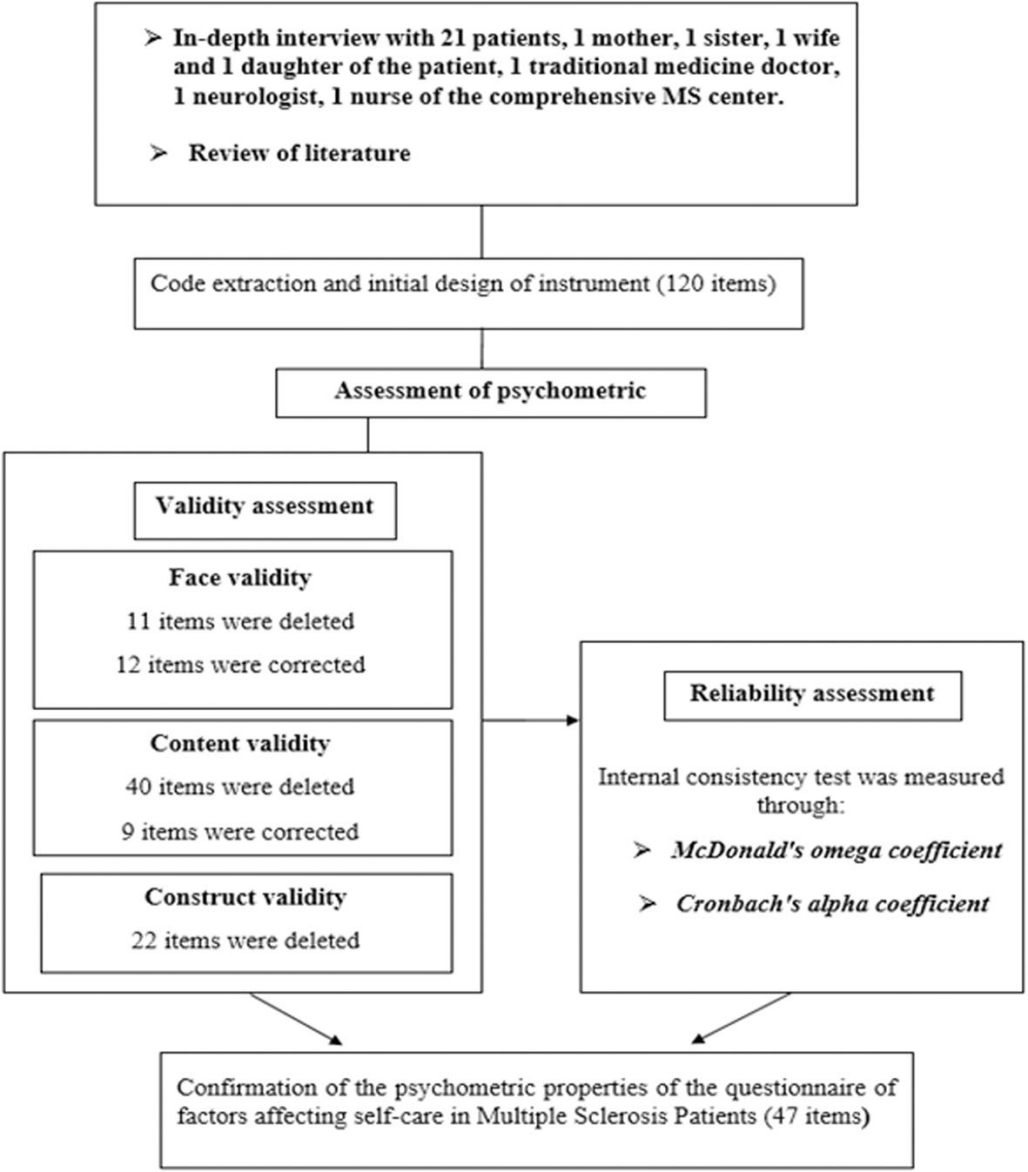
Schematic process of the reduction of the items of questionnaire

In this section, factors affecting self-care behavior in patients with multiple sclerosis were identified, which included four stages of understanding disease symptoms, tendency to conscious and targeted care, laziness in care and tendency to receive therapy services. In this stage, 120 questions were designed based on the qualitative stage and literature review (Fig. 1).

### Quantitative phase

#### Face validity

Based on the opinion of experts and a sample of the target group, 33 questions were corrected in the qualitative formal validity stage. In the quantitative face validity section, 11 questions were deleted and 9 questions were corrected. Finally, 109 questions remained in this part and entered the content validity stage (Fig. 1).

#### Content validity

Based on the opinion of experts, 18 questions were corrected in the validity of qualitative content. In the quantitative content validity section, 40 questions were deleted and 9 questions were corrected. In the end, 69 questions remained in this section and entered the construct validity (Fig. 1). For each question, CVR and CVI were calculated.

#### Construct validity

CFA method was used to check the construct validity of the questionnaire. At this stage, the questions that had a lower regression coefficient were removed to achieve an acceptable model.

The results of the CFA analysis showed that the value of the CR critical ratio in each question is higher than 1.96 and the significance level is < 0.001. The model fit indices for four subscales had standard values. The values of the X2/df, CFI, TLI, RMSEA indices were 2.31, 0.935, 0.920 and 0.051 respectively. The values of these indices confirmed the acceptability of the model (Fig. 1).

**Fig. 2:**
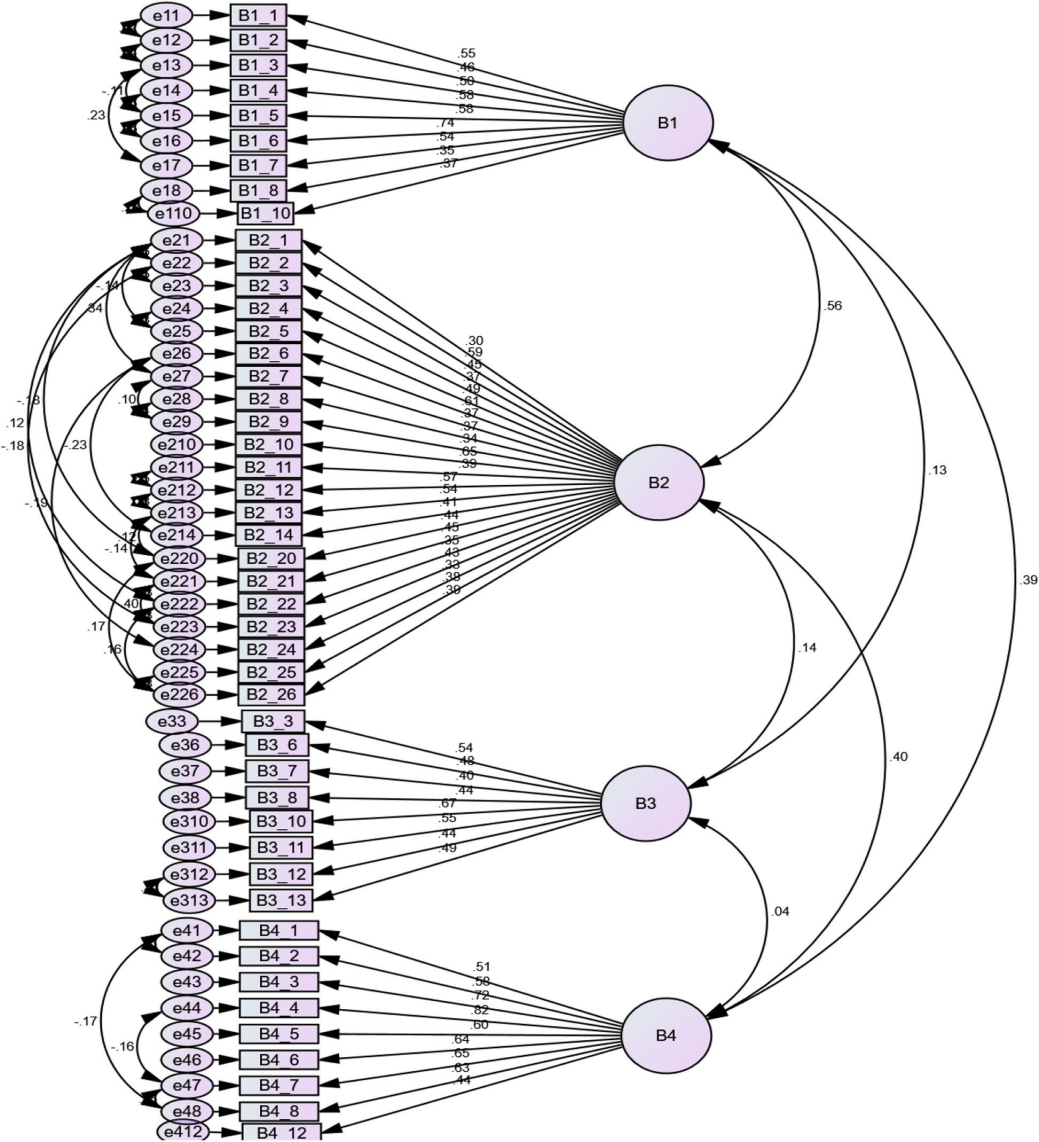
Confirmatory factor analysis diagram of the tool using Amos software

Finally, the questionnaire was confirmed with 47 questions and 4 subscales of understanding disease symptoms (9 questions), tendency to conscious and targeted care (21 questions), laziness in care (8 questions) and tendency to receive therapy services (9 questions) (Fig. 2, Table 2).

**Table 2:** Factors, items and factor loadings of the questionnaire of factors affecting self-care in multiple sclerosis patients (QFASMS)

Subscales	Items	Factor loadings	CVR	CVI
B1:Understanding the disease symptoms	Q1: The manifestation of disease symptoms, such as double vision and blurred vision, is important in adopting self-care behaviors	0.516	1	1
	Q2: The manifestation of disease symptoms, such as dizziness, numbness, and Speech disorders, are important in adopting self-care behaviors	0.459	1	1
	Q3: Errors in doing tasks, slowing of movements and slowing down in doing things are important in adopting self-care behaviors	0.502	1	1
	Q4: Neglecting initial symptoms leads to the progression of the disease	0.578	0.8	1
	Q5: Ignoring the symptoms of the disease delays taking care measures	0.575	0.8	0.90
	Q6: Accepting the existence of the disease is crucial in taking effective diagnostic measures to provide care	0.736	0.8	0.91
	Q7: To obtain information about an illness, doctors can provide suitable information	0.544	0.8	0.96
	Q8: Economic status is effective in adopting diagnostic measures	0.346	0.8	0.91
	Q9: The support and understanding of those around you is effective in taking diagnostic measures	0.364	1	1
B2: Tendency to conscious and targeted care	Q10: Accepting the disease of MS and understanding its chronicity is effective in controlling it	0.301	0.8	0.91
	Q11: The use of warm-natured foods can be effective in controlling MS disease	0.593	1	1
	Q12: The use of strengthening and complementary medicines can be effective in controlling the disease	0.452	0.6	0.91
	Q13: To control the illness, I use water with a balanced temperature for bathing	0.366	0.8	0.91
	Q14: Practicing yoga and meditation help to keep the illness under control	0.487	0.8	0.91
	Q15: I control my illness by listening to motivational messages, reading books, and attending various classes	0.609	0.6	0.78
	Q16: To prevent my condition from worsening, I refrain from getting hungry	0.368	0.6	0.80
	Q17: I decide what treatment methods to use	0.369	0.8	0.91
	Q18: I walk in nature to control my illness	0.335	0.6	0.80
	Q19: The ability to control thoughts and emotions is important in preventing the recurrence of illness	0.645	1	1
	Q20: I control my anger in the face of violent situations	0.391	0.6	0.91
	Q21: I avoid stressful situations to control my illness	0.574	0.6	0.91
	Q22: Due to the effect of the complications on reduction of sexual desires, I perform the relevant care and treatment measures	0.544	1	1
	Q23: To control the disease, I use the supplements recommended by the doctor, such as vitamin D, etc	0.411	1	0.93
	Q24: I follow the doctor's recommendations regarding medication use	0.440	0.6	0.78
	Q25: I think positively about my illness	0.453	1	0.96
	Q26: In order not to be a burden, I take care measures	0.349	1	1
	Q27: I avoid crowded places	0.429	0.6	0.80
	Q28: Using television and radio programs, you can obtain useful care informations	0.331	1	0.93
	Q29: By avoiding negative messages and news, I control my illness	0.383	0.6	0.96
	Q30: I have control over my thoughts	0.387	0.8	0.93
B3:Laziness in care	Q31: The difficulty of taking preventive measures is effective in reducing self-care motivation	0.543	0.8	1
	Q32: Confusion in receiving Medical services reduces self-care motivation	0.478	1	0.96
	Q33: The time-consuming nature of caregiving actions diminishes motivation for self-care	0.396	0.8	0.91
	Q34: The fact that some patients did not achieve any results from performing preventive measures reduces self-care motivation	0.438	0.6	0.78
	Q35: Family pressure is effective in taking preventive measures	0.669	0.8	0.91
	Q36: The pressure of illness is effective in taking preventive measures	0.552	0.8	0.90
	Q37: Medical costs are one of the barriers to self-care behaviors	0.439	1	1
	Q38: The high cost of sports makes self-care more difficult	0.489	0.6	0.80
B4:Tendency to receive therapy services	Q39: Decreasing care measures causes the disease to progress	0.512	1	0.96
	Q40: The feeling of drug dependence increases the tendency to receive medical services	0.583	0.8	0.91
	Q41: The prolongation of MS desease causes lifestyle changes	0.718	1	0.93
	Q42: Reducing attacks by taking therapeutic measures is important in the willingness to receive medical services	0.818	0.8	1
	Q43: To control the attacks of the disease, I resorted to therapeutic interventions	0.582	1	0.93
	Q44: The chronicity of the disease makes me diligent in receiving medicine	0.652	1	1
	Q45: The unknown and unclear complications of MS reduce the tendency to receive therapy services	0.636	1	0.96
	Q46: I have the ability to make decisions regarding the recommended treatment methods	0.632	0.6	0.91
	Q47: Fear and uncertainty about the effectiveness of therapeutic interventions delay the acceptance process	0.426	0.6	0.78

### Reliability

Cronbach's alpha coefficient for 4 subscales of understanding disease symptoms, tendency to conscious and targeted care, laziness in care and tendency to receive therapy services were equal to 0.777, 0.835, 0.724 and 0.846 respectively. McDonald's omega coefficient for 4 subscales of understanding disease symptoms, tendency to conscious and targeted care, laziness in care and tendency to receive therapy services were equal to 0.774, 0.833, 0.722 and 0.848, respectively. Cronbach's alpha and McDonald's omega index for all questions were 0.877 and 0.881, respectively (Table 3). In this study, ICC = 0.83 with 95% confidence interval ranges between 0/79 and 0.89 show that reliability is “good”.

**Table 3:** Cronbach’s alpha coefficients table by scales

Subscales	Item	Range coefficients	Cronbach’s alpha	McDonald’s omega coefficients
B1:Understanding disease symptoms	9	9–45	0.777	0.774
B2: Tendency to conscious and targeted care	21	21–105	0.835	0.833
B3:Laziness in care	8	8–40	0.724	0.722
B4:Tendency to receive therapy services	9	9–45	0.846	0.848
Total QFASMS	47	47–235	0.877	0.881

## Discussion

The purpose of this research was to design and validation of a questionnaire on the factors influencing self-care behaviors in patients with Multiple sclerosis. Studies have shown that the design of reliable tools is effective in better evaluating the effectiveness of interventions, better understanding the needs and expectations of patients, and better treatments [27]. This tool contains of 47 items in four dimensions (understanding disease symptoms, tendency to conscious and targeted care, laziness in care and tendency to receive therapy services) and is a valid and reliable scale. In this study, the grounded theory method was used to collect qualitative phase data.

Other designed self-care tools in chronic diseases, such as self-care questionnaires in diabetes patients [28] and the Scale of Perceptions and Self-Participation in Hemodialysis, also used Grounded Theory [29].

The tool items, covered factors affecting self-care behaviors in patients with multiple sclerosis. The validity and reliability of the scale were evaluated, and acceptable results for psychometrics were obtained. Other self-care tools, such as the Self-Care of Coronary Heart Disease Inventory Version 3 (SC-CHDI-V3) [30], Self-Care of Diabetes Inventory (SCODI) [31], and Self-Care of Heart Failure Index [32], used different validity and reliability methods for psychometrics.

To assess the validity of the questionnaire, face validity, content validity, and construct validity were used. CVR and CVI were calculated for all the questions. The Cronbach's alpha coefficient and McDonald's Omega coefficient for the entire questionnaire were 0.877 and 0.881, respectively. Finally, a questionnaire with 47 questions and 4 subscales of understanding the symptoms of the disease (9 questions), tendency to conscious and targeted care (21 questions), laziness in care (8 questions) and tendency to receive therapy services (9 question) was approved. Based on the results, if the CVR is greater than 0.6 and the CVI is greater than 0.78, then it is considered acceptable [33]. If the Cronbach's alpha value is above 0.7, it is considered suitable and indicates strong internal consistency among the questions [34].

The first subscale of this questionnaire was "Understanding Disease Symptoms." This subscale has been validated with 9 questions, CVR 0.8 to 1, CVI 0.90 to 1, regression coefficient (factor loading) 0.346 and 0.736, Cronbach's alpha 0.777, and McDonald's omega 0.774. Understanding the symptoms of the disease is understanding the occurrence of changes in the state and conditions of the body that indicate being affected by a disease and can be reported by the patient. The understanding of illness provides an important framework for investigating patients' beliefs and how its components influence health behaviors [35]. This dimension is present in all other self-care tools, indicating its importance [28, 36]. Intervention studies have shown that perceptions of illness can change [36]. Perception, interpretation, management, and communication of symptoms have a strong impact on healthcare utilization [37].

Based on the study of Moradi Mutlaq, the results of the confirmatory factor analysis of the questionnaire "perceived vulnerability to contagious diseases" showed that the questionnaire of 15 questions with 2 dimensions has an acceptable structure validity. The values of two important indices, χ2/df and RMSEA, were 1.68 and 0.05, respectively. This questionnaire has sufficient convergent validity and possesses Cronbach's alpha reliability coefficient of 0.83 and test-retest reliability [38]. 

The second subscale of this questionnaire was "tendency to conscious and targeted care". This subscale has been validated with 21 questions, CVR 0.6 to 1, CVI 0.78 to 1, regression coefficient (factor loading) 0.301 and 0.645, Cronbach's alpha 0.835, and McDonald's omega 0.833. The tendency to conscious and targeted care is the desire and internal tendency of a person to evaluate and consciously perform care behaviors to achieve the goal.

Based on Na'mati Zadeh's study, the results of CFA of the "Self-Care in Type 2 Diabetes" questionnaire showed that the 34-item questionnaire with 4 dimensions has an acceptable structural validity (χ2/df = 2.40; CFI = 0/91; TLI = 0/91; NFI = 0/86; GFI = 0/85; IFI = 0/91; AGFI = 0/82; RMSEA = 0/054; RFI = 0/85). The reliability results of the tool also showed that Cronbach's alpha coefficient is 0.95, which is acceptable [39]. Studies have shown that when a disease process occurs, some may make a conscious decision to take care of themselves [40]. Evidence shows that conscious self-care moderates many mental and physical consequences of chronic diseases and can help prevent and reduce the economic burden [41].

The third subscale of this questionnaire was "laziness in care ". This subscale has been validated with 8 questions, CVR 0.6 to 1, CVI 0.78 to 1, regression coefficient (factor loading) 0.396 and 0.669, Cronbach's alpha 0.724, and McDonald's omega 0.722. Based on the study conducted by Naderi et al., the results of the CFA of the questionnaire "Measurement of perceived barriers for self-care in middle-aged patients with diabetes mellitus type 2" demonstrated that the 23-item questionnaire with 7 dimensions has an acceptable structural validity (CFI = 0.93; IFI = 0.93; NNFI = 0.91; NFI = 0.85; RMSEA = 0.056). "The reliability of the tool also indicated a Cronbach's alpha coefficient of 0.80, which is acceptable. Test–retest of the scale with a 2-week interval indicated an appropriate stability for the scale (ICC = 0.89) [39]. Self-care is a vital strategy to improve the quality of life of patients with multiple sclerosis (MS) [8]. Neglecting self-care has serious consequences for the health and well-being the neglectful individuals and may even have consequences for society [42]. The results of studies show that patients with diabetes gradually experience a decrease in self-care. The causes of reduced care include individual, economic, social, educational, and psychological barriers [43]. Mansyur et al. showed that there is a relationship between norms and social barriers, social support, with adherence to self-care in diabetes patients. Thus, people who received less support faced more barriers and had lower levels of adherence to self-care [44].

The fourth subscale of this questionnaire was "tendency to receive therapy services". This subscale has been validated with 9 questions, CVR 0.6 to 1, CVI 0.80 to 1, regression coefficient (factor loading) 0.426 and 0.818, Cronbach's alpha 0.846, and McDonald's omega 0.848. Based on Whitney Scott's study, the results of the confirmatory factor analysis of the questionnaire "Confirmatory Factor Analysis of Facets of Psychological Flexibility in a Sample of People Seeking Treatment for Chronic Pain" showed that the 98-question questionnaire with 6 dimensions has acceptable construct validity (chi-square (522) = 2326.03, *p* < 0.001; RMSEA = 0.08; CFI = 0.94; TLI = 0.94). The reliability results of the tool also demonstrated an acceptable Cronbach's alpha coefficient of 0.92. The results of a study showed that chronic patients tend to follow treatment measures and change their lifestyle [45].

Dashti et al. showed in a study that intervention programs effectively improve health-promoting behaviors in patients with various diseases [46]. Robatsarpooshi et al. showed that the design of self-care tools can measure the reasons for performing or not performing self-care behaviors in patients. Determining the level and stage of the patient's self-care, determining suitable intervention solutions to solve the problems and obstacles of patients in adopting self-care behavior, are possible by completing the questionnaire [28].

The study provides a new instrument to measure and capture factors affecting self-care behaviors in people with MS, but however may need proper translation and cultural adaptation to scientifically evaluate its usage in different MS populations. Cultural adaptation is a process in which any differences between the source culture and the target culture are taken into account in order to maintain equivalence in meaning [47].

One of the limitations of this study was the concern of the patients about completing the questionnaire, which was solved by providing explanations about the objectives of the study.

## Conclusions

A valid tool for measuring change of factors influencing self-care behaviors in patients with Multiple sclerosis and, that this tool may be guiding the development of effective programs that regard self-care behaviours in MS. I am not sure that such programs would prevent the progression of MS, but rather the ability of persons with MS to manage and self-monitor MS in daily life. That would hypothetically promote quality of life and wellbeing, and overall functioning with a chronic neurological disease. This needs to be scientifically evaluated in research studies with experimental designs.

### Supplementary Information

**Additional file 1.** Demographic questionnaires and factors affecting self-care of multiple sclerosis patients (QFASMS) were used to collect data.

## Data Availability

The data sets used and/or analyzed during the current study was available from the corresponding author on reasonable request.

## Abbreviation

CNS: Central Nervous System
MS: Multiple sclerosis
CVR: Content validity ratio; CVI: Content validity index; CFA: Confirmatory factor analysis; QFASMS: Questionnaire of Factors Affecting Self-Care in Multiple Sclerosis Patients
× ^2^/df: Chi-square ratio to the degree of freedom
CFI: Comparative fit index
RMSEA: Root means the square error of approximation
TLI: Tucker-Lewis index
ICC: Interclass correlation coefficients
NCP: Non-centrality parameter
