# Supplementary material for: Design and validation of a Questionnaire on the factors influencing self-care behaviors in patients with Multiple sclerosis (QFASMS)
Source: BMC Neurol. 2024 Jan 4;24:20. doi: 10.1186/s12883-023-03522-x (PMC10765624; doi:10.1186/s12883-023-03522-x)
Supplement: Supplementary file 1 — Additional file 1. Demographic questionnaires and factors affecting self-care of multiple sclerosis patients (QFASMS) were used to collect data. [file 12883_2023_3522_MOESM1_ESM.docx]

**Demographic questionnaires and factors affecting self-care of multiple sclerosis patients (QFASMS) were used to collect data.**

**This questionnaire was approved by the ethics committee of Mashhad University of Medical Sciences with the ethics code IR.MUMS. FHMPM.REC.1400.024.**

| **background/demographic variables:** | |
| --- | --- |
| Age: ......... Year........... Month .............. Day | Gender: Male 󠇧󠇧 󠇧 Female󠇧 󠇧󠇧 |
| Occupation: retired 󠇧󠇧 employee 󠇧󠇧 free 󠇧󠇧 housewife 󠇧󠇧 unemployed 󠇧󠇧 worker 󠇧󠇧 other 󠇧󠇧 | |
| education level: Doctorate degree 󠇧󠇧 Master's degree 󠇧 󠇧󠇧 Bachelor's degree 󠇧󠇧 Associate Degree󠇧 󠇧󠇧  diploma 󠇧󠇧 Incomplete diploma󠇧 󠇧󠇧 illiterate󠇧 󠇧󠇧 | |
| Marital status: single 󠇧󠇧 married 󠇧󠇧 separated from spouse/divorced 󠇧󠇧 deceased spouse 󠇧󠇧 | |
| Duration of MS disease: ..............................years | Age of onset of MS: .....................years |

| strongly agree | agree | neither agree nor disagree | disagree | strongly disagree | Items | Subscales |
| --- | --- | --- | --- | --- | --- | --- |
|  |  |  |  |  | Q1: The manifestation of disease symptoms, such as double vision and blurred vision, is important in adopting self-care behaviors. | **Understanding the disease symptoms** |
|  |  |  |  |  | Q2: The manifestation of disease symptoms, such as dizziness, numbness, and Speech disorders, are important in adopting self-care behaviors. |  |
|  |  |  |  |  | Q3: Errors in doing tasks, slowing of movements and slowing down in doing things are important in adopting self-care behaviors. |  |
|  |  |  |  |  | Q4: Neglecting initial symptoms leads to the progression of the disease. |  |
|  |  |  |  |  | Q5: Ignoring the symptoms of the disease delays taking care measures. |  |
|  |  |  |  |  | Q6: Accepting the existence of the disease is crucial in taking effective diagnostic measures to provide care. |  |
|  |  |  |  |  | Q7: To obtain information about an illness, doctors can provide suitable information. |  |
|  |  |  |  |  | Q8: Economic status is effective in adopting diagnostic measures. |  |
|  |  |  |  |  | Q9: The support and understanding of those around you is effective in taking diagnostic measures. |  |
|  |  |  |  |  | Q10: Accepting the disease of MS and understanding its chronicity is effective in controlling it. | **Tendency to conscious and targeted care** |
|  |  |  |  |  | Q11: The use of warm-natured foods can be effective in controlling MS disease. |  |
|  |  |  |  |  | Q12: The use of strengthening and complementary medicines can be effective in controlling the disease. |  |
|  |  |  |  |  | Q13: To control the illness, I use water with a balanced temperature for bathing. |  |
|  |  |  |  |  | Q14: Practicing yoga and meditation help to keep the illness under control. |  |
|  |  |  |  |  | Q15: I control my illness by listening to motivational messages, reading books, and attending various classes. |  |
|  |  |  |  |  | Q16: To prevent my condition from worsening, I refrain from getting hungry. |  |
|  |  |  |  |  | Q17: I decide what treatment methods to use. |  |
|  |  |  |  |  | Q18: I walk in nature to control my illness. |  |
|  |  |  |  |  | Q19: The ability to control thoughts and emotions is important in preventing the recurrence of illness. |  |
|  |  |  |  |  | Q20: I control my anger in the face of violent situations. |  |
|  |  |  |  |  | Q21: I avoid stressful situations to control my illness. |  |
|  |  |  |  |  | Q22: Due to the effect of the complications on reduction of sexual desires, I perform the relevant care and treatment measures. |  |
|  |  |  |  |  | Q23: To control the disease, I use the supplements recommended by the doctor, such as vitamin D, etc. |  |
|  |  |  |  |  | Q24: I follow the doctor's recommendations regarding medication use. |  |
|  |  |  |  |  | Q25: I think positively about my illness. |  |
|  |  |  |  |  | Q26: In order not to be a burden, I take care measures. |  |
|  |  |  |  |  | Q27: I avoid crowded places. |  |
|  |  |  |  |  | Q28: Using television and radio programs, you can obtain useful care in formations. |  |
|  |  |  |  |  | Q29: By avoiding negative messages and news, I control my illness. |  |
|  |  |  |  |  | Q30: I have control over my thoughts. |  |
|  |  |  |  |  | Q31: The difficulty of taking preventive measures is effective in reducing self-care motivation. | **Laziness in care** |
|  |  |  |  |  | Q32: Confusion in receiving Medical services reduces self-care motivation. |  |
|  |  |  |  |  | Q33: The time-consuming nature of caregiving actions diminishes motivation for self-care. |  |
|  |  |  |  |  | Q34: The fact that some patients did not achieve any results from performing preventive measures reduces self-care motivation. |  |
|  |  |  |  |  | Q35: Family pressure is effective in taking preventive measures. |  |
|  |  |  |  |  | Q36: The pressure of illness is effective in taking preventive measures. |  |
|  |  |  |  |  | Q37: Medical costs are one of the barriers to self-care behaviors. |  |
|  |  |  |  |  | Q38: The high cost of sports makes self-care more difficult. | **Tendency to receive therapy services** |
|  |  |  |  |  | Q39: Decreasing care measures causes the disease to progress. |  |
|  |  |  |  |  | Q40: The feeling of drug dependence increases the tendency to receive medical services. |  |
|  |  |  |  |  | Q41: The prolongation of MS disease causes lifestyle changes. |  |
|  |  |  |  |  | Q42: Reducing attacks by taking therapeutic measures is important in the willingness to receive medical services. |  |
|  |  |  |  |  | Q43: To control the attacks of the disease, I resorted to therapeutic interventions. |  |
|  |  |  |  |  | Q44: The chronicity of the disease makes me diligent in receiving medicine. |  |
|  |  |  |  |  | Q45: The unknown and unclear complications of MS reduce the tendency to receive therapy services. |  |
|  |  |  |  |  | Q46: I have the ability to make decisions regarding the recommended treatment methods. |  |
|  |  |  |  |  | Q47: Fear and uncertainty about the effectiveness of therapeutic interventions delay the acceptance process. |  |
